# Heat stroke dysfunctions: from pathophysiology to prediction

**DOI:** 10.3389/fphys.2025.1700342

**Published:** 2025-12-02

**Authors:** Azza Alawad, Tarig Merghani, Nadia Yousif, Shahenaz Satti, Alhiedi Edris, Alwaleed Hakim, Tarig Fadelelmoula

**Affiliations:** 1 Department of Physiology, Faculty of Medicine, Al Neelain University, Khartoum, Sudan; 2 Department of Physiology, RAK College of Medical Sciences, RAK Medical and Health Sciences University, Ras Al Khaimah, United Arab Emirates; 3 Department of Pediatrics, Ministry of Health, Khartoum, Sudan; 4 Department of Physiology, College of Medicine and Health Sciences/National University of Science and Technology, Sohar, Oman; 5 Zaeyd Military Hospital, Ministry of Health, Abu Dhabi, United Arab Emirates; 6 Faculty of Medicine, Alexandria University, Alexandria, Egypt; 7 Department of Internal Medicine, College of Medicine and Health Sciences/National University of Science and Technology, Sohar, Oman

**Keywords:** heat stroke, hyperthermia, thermoregulation, inflammation, intestinal microbiota, multiple organ failure, artificial intelligence, wearable devices

## Abstract

Heat stroke is a severe, life-threatening medical emergency defined by an elevation in core body temperature exceeding 40.0 °C, accompanied by acute central nervous system (CNS) dysfunction and often complicated by multi-organ failure. Although traditionally viewed as a thermoregulatory collapse from environmental exposure or intense exertion, recent evidence highlights its complex, multifactorial pathophysiology. This includes systemic inflammation, immune dysregulation, oxidative stress, endothelial injury, and activation of the coagulation cascade. This comprehensive narrative examines advances in understanding underlying mechanisms, clinical manifestations, emerging biomarkers, and outcomes in both classic (non-exertional) and exertional heat stroke. Emphasis is placed on the gut–brain axis, where disruption of intestinal barrier integrity and microbiota dysbiosis amplify systemic inflammation and contribute to neurotoxicity. Heat stroke-related neurological damage affects critical brain regions, including the hypothalamus, cerebellum and hippocampus, often resulting in long-term cognitive and motor impairments. Several biomarkers that include interleukin-6 (IL-6), high-mobility group box 1 protein (HMGB1), creatine kinase (CK), S100β, and D-dimer are under active investigation for diagnostic and prognostic utility, but their clinical use remains limited by inter-individual variability and lack of standardized thresholds. Recent advances in artificial intelligence (AI) and wearable biosensors may facilitate early detection, continuous monitoring, and individualized risk prediction, particularly in vulnerable populations such as outdoor workers, athletes, and military personnel. An interdisciplinary approach is critical to improving early recognition, management strategies, and long-term outcomes in the context of rising global temperatures and climate change.

## 1 Introduction

Heat stroke is a severe, life-threatening medical condition characterized by a core body temperature exceeding 40.0 °C (104°F), accompanied by central nervous system dysfunction, and often complicated by multi-organ injury (Boucha et al., 2002). It is the most severe form of heat-related illnesses, requiring urgent intervention to prevent irreversible damage or death (Jain et al., 2018; Keatinge, 2003). Heat stroke has two main forms: classic (non-exertional), which affects vulnerable individuals (children, older adults or chronically ill patients) during heatwaves, and exertional, which occurs in otherwise healthy individuals engaged in strenuous physical activity in high-temperature environments (Abderrezak et al., 2022). Despite differing causes, both share similar clinical features, including systemic inflammation, tissue damage, and neurological effects (Laitano et al., 2019a).

With the accelerating pace of climate change, the global incidence of heat stroke is rising (Khan and Mubeen, 2025), affecting broader groups (outdoor workers and pregnant women) and highlighting the need for better public health measures, early detection, and deeper insight into its underlying mechanisms. According to the World Health Organization (WHO), heat-related illnesses, including heat stroke, are among the most serious health threats exacerbated by climate change (World Health, 2024). Consistent with this concern, recent epidemiological data underscore the growing burden of heat stroke. In 2023, the U.S. Armed Forces reported a crude incidence rate of 31.7 cases of heat stroke and 172.7 cases of heat exhaustion per 100,000 person-years, noting a decline in heat stroke incidence but a rise in heat exhaustion over the 2019–2023 surveillance period (Maule et al., 2024). In the general population, earlier studies estimated about 1.34 emergency department visits per 100,000 people annually for heat stroke in the United States (Hess et al., 2014). Although the incidence of heat stroke varies over time, mortality rates remain significant. In-hospital case fatality is estimated at approximately 3.5%; however, this figure likely underrepresents the true burden, as it excludes pre-hospital deaths and cases that are misdiagnosed (Cong et al., 2025). Recent research indicates that official mortality records substantially underestimate the association between heat exposure and heat-related deaths (Longden et al., 2020). Additionally, the broader mortality burden linked to rising ambient temperatures has increased, with a previous study showing that heat-related excess deaths grew from 0.83% in 2000–2003 to 1.04% in 2016–2019 (Zhao et al., 2021). These findings collectively highlight the growing impact of climate change on heat-related morbidity and mortality, reinforcing the need for improved surveillance, prevention, and early intervention strategies.

Despite clinical awareness of heat stroke among physicians and scientists, the pathophysiological mechanisms underlying its progression remain incompletely understood. Historically, heat stroke was viewed primarily as a thermoregulatory failure resulting from environmental or exertional heat overload (Iba et al., 2022). However, emerging research suggests that heat stroke is not merely a thermal disorder but a systemic inflammatory condition with features resembling sepsis (Lim, 2018). Elevated core temperature initiates a cascade of cellular and molecular responses involving heat shock proteins, cytokine storms, oxidative stress, endothelial dysfunction, and coagulation abnormalities (Iba et al., 2022; Lim, 2018; Iba et al., 2023a). These mechanisms contribute to both short-term and long-term multi-organ damage.

In recent years, growing attention has been given to the involvement of the gut-brain axis and intestinal microbiota in the development and progression of heat stroke (Wen et al., 2021). The rise in body temperature can compromise integrity of intestinal barrier, allowing endotoxins and bacterial components to enter systemic circulation (Hosseindoust et al., 2022). Animal studies showed that microbial translocation promotes inflammatory response and contributes to multi-organ damage and could be linked to post-heat stroke alterations in cognitive function (Cao et al., 2021; Khakisahneh et al., 2020). These insights mark a paradigm shift from viewing the gastrointestinal system as a passive target of heat injury to an active participant in disease progression.

Another critical area of concern is the effect of heat stroke on the central nervous system. While acute manifestations such as delirium, seizures, ataxia, and coma are well-recognized and may be resolved with timely treatment, growing evidence indicates that neurological injury can persist long after recovery (Lawton et al., 2019; Adnan Bukhari, 2023; Asmara, 2020). Survivors frequently experience lasting cognitive, emotional, and motor impairments, including memory loss, executive dysfunction, personality changes, and coordination difficulties (Lawton et al., 2019; Adnan Bukhari, 2023; As and mara, 2020). These effects result from several pathological mechanisms, including direct damage to neurons, breakdown of the blood brain barrier, and ongoing inflammation in the brain (Christogianni et al., 2018). Imaging and histological studies have shown that heat exposure can injure sensitive areas such as the cerebellum, hippocampus, and hypothalamus through processes like cytokine-induced toxicity, oxidative stress, and reduced blood flow (He et al., 2023). Deeper understanding of these alterations may support the development of neuroprotective and rehabilitative strategies to mitigate long-term disability. Given the complexity and variability in clinical presentation and outcomes, the identification of reliable biomarkers for early detection, risk stratification, and prognosis is critical (Schlader et al., 2022). Many biomarkers such as creatine kinase (CK), high-sensitivity troponin I (hs-TnI), interleukin-6 (IL-6), D-dimer, S100B protein, high mobility group box 1 (HMGB1), thrombin–antithrombin III complex (TAT), syndecan-1, and von Willebrand factor antigen (vWfAg) are under investigation for their potential to support clinical decision-making and enhance patient outcomes (Zhao et al., 2021; Schlader et al., 2022). Furthermore, advancements in artificial intelligence and machine learning offer promising tools for heat stroke prediction, diagnosis, and management. These technologies can analyze environmental data, physiological signals, and clinical parameters to identify individuals at risk and enable timely interventions (Son et al., 2021; Kiyatkin and Sharma, 2009).

This narrative review synthesizes current knowledge on heat stroke based on peer-reviewed articles, clinical guidelines, case reports, and reviews. References were identified through electronic databases including PubMed, Scopus, and Google Scholar using search terms such as “heat stroke,” “hyperthermia,” “coagulopathy,” and “gut–brain axis.” Additional sources were identified from the bibliographies of relevant publications. As a narrative review, this work does not adhere to systematic review protocols but instead offers a conceptual synthesis of key findings across disciplines. Through this work we aim to consolidate emerging knowledge on the diverse and evolving aspects of heat stroke. We begin by classifying its clinical forms and then explore recent developments in the understanding of pathophysiological mechanisms, with emphasis on inflammatory cascades, thermoregulatory center dysfunction, and gut–brain axis involvement. Furthermore, we discuss neurological consequences, current biomarker research, and the transformative potential of artificial intelligence in addressing this growing health crisis.

## 2 Methods

Major electronic databases including MEDLINE/PubMed, Scopus, Web of Science, and Google Scholar were searched for this review. Key search terms included combinations of “heat stroke,” “exertional heat stroke,” “classic heat stroke,” “pathophysiology,” “clinical types,” “epidemiology,” “management,” “prevention,” “gut–brain axis,” “intestinal permeability,” “microbiota,” “wearable devices,” “biosensors,” and “physiological monitoring.” Publicly available reports from recognized authorities (e.g., WHO, CDC) were also consulted when relevant. Titles and abstracts retrieved from all searches were screened for relevance; non-English publications, commentaries without original analysis, and articles unrelated to heat-related illness were excluded. Peer-reviewed original studies, systematic reviews, and meta-analyses addressing epidemiology, pathophysiology, clinical features, early detection, prevention, and management of heat stroke were selected. Three authors independently screened titles and abstracts, then they assessed full texts for eligibility, and extracted data with disagreements resolved by discussion. Findings were synthesized narratively under thematic the following domains: Clinical types, Emerging concepts in the pathophysiology, Neurological effects, Biomarkers for early detection and prognosis, Artificial intelligence Applications in heat stroke prediction and diagnosis, and Management of heat stroke).

## 3 Clinical types of heat stroke

Heat stroke is categorized into two main types: Classic Heat Stroke (CHS) and Exertional Heat Stroke (EHS), which differ in cause, population affected, clinical features, and outcomes (Laitano et al., 2019b). Classic heat stroke (CHS) usually occurs after prolonged exposure to high environmental temperatures and humidity without significant physical activity. It primarily affects children and older adults, particularly those with underlying medical conditions or impaired ability to regulate body temperature (Laitano et al., 2019b; Yezli et al., 2023). In contrast, EHS results from intense physical exertion in hot environments, affecting primarily young individuals, such as athletes, military personnel, and outdoor workers (Laitano et al., 2019b; Périard et al., 2022). The onset of symptoms in CHS is usually gradual, whereas in EHS, it tends to be sudden and rapid (Laitano et al., 2019b; Yezli et al., 2023; Périard et al., 2022). Another key distinction is sweating; it is often absent or minimal in CHS, while initially present in EHS but insufficient and may diminish as the condition progresses (Laitano et al., 2019b; Yezli et al., 2023; Périard et al., 2022). In classic heat stroke (CHS), the main underlying mechanism is reduced heat loss resulting from failure of the body’s thermoregulatory system. In contrast, exertional heat stroke (EHS) is driven by excessive internal heat production during intense physical activity, which overwhelms the body’s ability to dissipate heat (Laitano et al., 2019b; Yezli et al., 2023; Périard et al., 2022). CHS tends to have higher rates of mortality and complications, largely due to delayed recognition and treatment, whereas EHS generally has a more favorable outcome when managed promptly (Belval et al., 2018).

**FIGURE 1:**
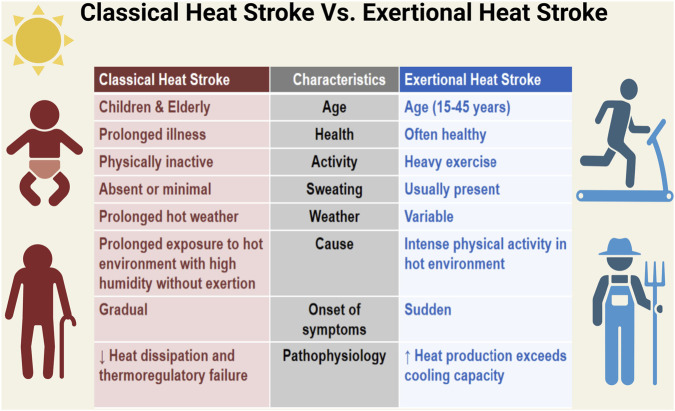
Comparison between classic and exertional heat stroke across multiple clinical and physiological dimensions. Data illustrated in this figure are derived from human clinical studies. Created with BioRender.com.

The following figure presents key differences in epidemiology, clinical features, and underlying mechanisms that help to distinguish between the two forms of heat stroke in various populations (Figure 1).

## 4 Pathophysiology of heat stroke

### 4.1 Emerging concepts

Recent insights into the pathophysiology of heat stroke reveal a multifaceted process involving: (i) Hypothalamic thermoregulatory failure and systemic consequences (ii) Systemic inflammation (iii) Coagulation disturbances (iv) Gut-Derived Immune Activation and Microbiota Dysregulation, and (v) Neuroinflammation. These overlapping mechanisms underscore the complexity of heat stroke, which changes rapidly from cellular stress to multi-organ failure, with both acute and long-term neurological and systemic implications.

#### 4.1.1 Hypothalamic thermoregulatory failure and systemic consequences

The thermoregulatory center, located in the preoptic area of the anterior hypothalamus, plays a pivotal role in maintaining core body temperature within a narrow physiological range (∼37 °C ± 0.5 °C). It receives signals from both peripheral and central thermoreceptors and coordinates autonomic, hormonal, and behavioral responses to maintain thermal balance. The anterior hypothalamus promotes heat loss through mechanisms such as vasodilation and sweating, while the posterior hypothalamus facilitates heat gain by inducing vasoconstriction, shivering, and increased metabolic activity.

In cases of heat stroke, prolonged exposure to high ambient temperatures or intense physical exertion can overwhelm the regulatory mechanisms, leading to failure of heat loss pathways, such as sweating and cutaneous vasodilation. As core temperature continues to rise, direct thermal injury to the hypothalamus may occur, impairing its ability to activate compensatory responses (Vornanen, 2020). This damage disrupts sympathetic output, suppresses evaporative cooling, and contributes to systemic complications such as multi-organ dysfunction, cerebral edema, and coagulopathy (Vornanen, 2020; Bich et al., 2025). Moreover, reduced cerebral perfusion due to cardiovascular strain and dehydration may exacerbate hypothalamic injury (Périard et al., 2021). Without prompt intervention, this failure of thermoregulation can result in irreversible central nervous system damage and death.

Under normal conditions, thermoregulation is remarkably effective, maintaining core body temperature within a narrow range despite wide fluctuations in ambient temperature. Compensatory mechanisms such as increased cardiac output, splanchnic vasoconstriction, and sweating facilitate heat dissipation. At the cellular level, exposure to elevated temperatures triggers the expression of heat shock proteins (HSPs), which play a crucial role in protecting cells from heat-induced injury (Coombs, 2020).

However, when environmental stress exceeds physiological limits, the thermoregulatory mechanisms are no longer sufficient. For instance, when environmental humidity exceeds 75%, evaporation of sweating is markedly reduced, limiting its cooling effect. In addition, alternative heat loss mechanisms, such as radiation, conduction, and convection, become inadequate when ambient temperature exceeds skin temperature. Under such conditions, the body becomes unable to maintain thermal balance, leading to rapid increase in core body temperature and higher risk of cellular and systemic complications (Koop and Tadi, 2025).

One of the earliest systemic consequences is dehydration, due to both excessive sweating and reduced fluid intake. This leads to volume depletion and electrolyte disturbances such as hypernatremia or normo-natremic dehydration (Lipman et al., 2021). In rare cases, excessive intake of hypotonic fluids may result in hyponatremia, particularly in exertional heat stroke cases such as marathon runners (Klingert et al., 2022). Additionally, hyperkalemia may arise due to muscle breakdown (particularly in rhabdomyolysis) or acidosis, leading to a shift of potassium from cells into plasma resulting in cardiovascular and renal complications (Laitano et al., 2020). Hypocalcemia may also occur, and when combined with hyperkalemia, may cause dangerous ECG abnormalities and potentially fatal arrhythmias (Leiva and Church, 2025).

**FIGURE 2:**
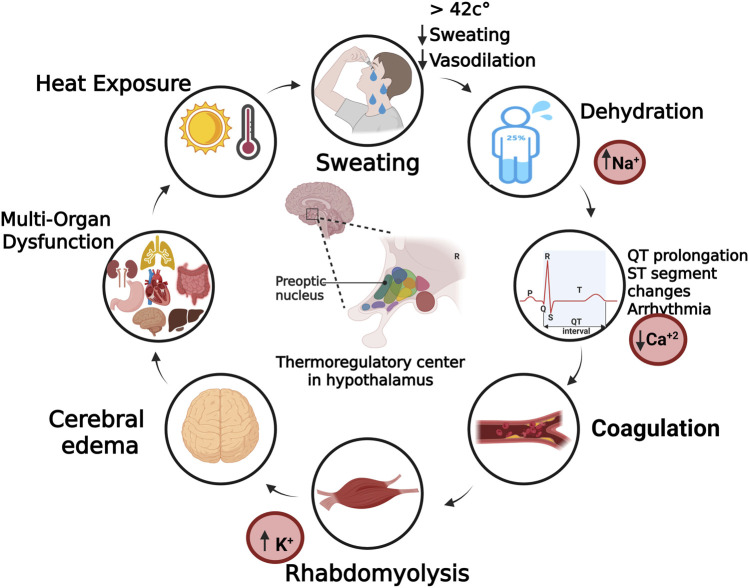
Systemic consequences of heat stress. Data illustrated in this figure are derived from both human clinical studies and supporting animal research models. Created with BioRender.com.

Other abnormalities such as coagulopathies, ranging from mild coagulation disturbances to severe disseminated intravascular coagulation (DIC), along with cerebral edema and direct neuronal damage are major consequences of heat stroke (Iba et al., 2023b). As these abnormalities progress, they disrupt homeostatic processes and impair organs functions resulting in multi-organ failure. Figure 2 illustrates the cascade of systemic effects that contribute to multi-organ dysfunction following disruption of thermoregulatory mechanisms under extreme heat exposure.

#### 4.1.2 Systemic inflammation

Recent studies have broadened our understanding of the complex inflammatory and molecular mechanisms underlying heatstroke. These responses involve systemic immune activation and endothelial dysfunction, all of which contribute to tissue injury, vascular leakage, and the progression to multi-organ failure (Iba et al., 2023a; Yu et al., 2024). Hyperthermia triggers a coordinated stress response involving endothelial, leukocyte, and epithelial cells, regulated by heat-shock proteins (HSP) (Leon and Bouchama, 2015). HSP including HSP70 play a pivotal role in reducing inflammation and preserving protein homeostasis within the cells (Hu et al., 2022). Activated endothelial cells release syndecan-1, von Willebrand factor, and endothelin-1, reflecting vascular injury and correlating with disease severity (Wang et al., 2025). Simultaneously, epithelial barrier damage and leukocyte activation promote cytokine release and oxidative stress, amplifying inflammation and tissue damage. This leads to altered levels of inflammatory cytokines in plasma and tissues (Yan et al., 2006). In case of prolonged hyperthermia, the immune, epithelial and endothelial responses become accelerated, causing hypoxia, circulatory failure, and increased metabolic derangement. A key mediator in this inflammatory cascade is high mobility group box 1 (HMGB1), a nuclear protein that acts as a damage-associated molecular pattern (DAMP) (Zhang et al., 2024). Once released extracellularly, HMGB1 acts as an indicator that binds to pattern recognition receptors such as Toll-like receptor 4 (TLR4) and the receptor for advanced glycation end-products (RAGE), activating NF-κB signaling and promoting the release of proinflammatory cytokines (Yang et al., 2002). The resulting cytokine surge that includes interleukin (IL)-1α, IL-1β, IL-6, IL-8, IL-10, IL-12, interferon-gamma (IFN-γ), tumor necrosis factor-alpha (TNF-α), and soluble TNF receptors (sTNFR) and others further recruits and activates leukocytes, leading to cell death via apoptosis, necroptosis, and neutrophil extracellular trap formation (NETosis), which intensifies tissue injury and sustains inflammation resulting in multi-organ dysfunction (Leon and Helwig, 2010).

#### 4.1.3 Coagulation disturbances

In parallel to the inflammation, immune cells contribute to the development of a procoagulant state through the expression of tissue factor (TF) and activation of coagulation factor VIIa, which generates thrombin and initiates disseminated intravascular coagulation (DIC) (Popescu et al., 2022). DIC can be hyper-fibrinolytic coagulation (associated with thrombotic events), or hypofibrinolytic (associated with excessive bleeding). Although the exact triggers are difficult to determine, it is assumed that endothelial injury impairs fibrinolysis via upregulation of plasminogen activator inhibitor-1 (PAI-1), alongside a reduction in natural anticoagulants such as antithrombin and protein C (Zhao et al., 2021; Iba et al., 2023a). Additionally, the release of von Willebrand factor (VWF) promotes platelet adhesion and aggregation, driving the formation of inflammatory thrombi. The convergence of systemic inflammation and widespread micro-thrombosis culminates in DIC (hyper or hypofibrinolytic). Clinical findings often include elevated fibrin degradation products, prolonged prothrombin time (PT) and partial thromboplastin time (aPTT), and in severe scenarios, life-threatening events such as intracerebral hemorrhage (Zhao et al., 2021; Iba et al., 2023a; Zhang et al., 2020).

**FIGURE 3:**
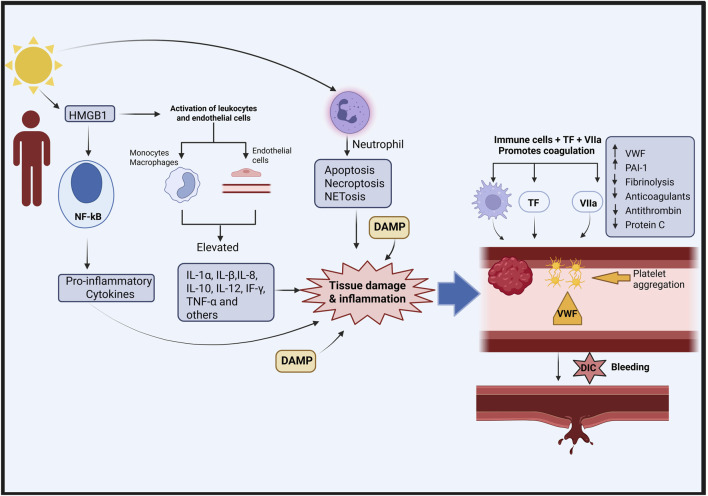
Pathophysiological sequence of heat stroke. Pathway steps illustrated are supported by human clinical studies and complementary experimental animal research. Created with BioRender.com.

The cascade of pathological events in heat stroke is depicted in Figure 3, which outlines a progression initiated by immune system activation and excessive cytokine release leading to inflammation, endothelial injury, and coagulation disturbances.

#### 4.1.4 Gut-derived immune activation and microbiota dysregulation

During heat stroke and when the core body temperature rises, severe injury to both the small and large intestines may occur, with significant loss of intestinal epithelial cells (Ogden et al., 2020). In addition to hyperthermia, intestinal injury may be caused by hypoperfusion and oxidative stress (Dokladny et al., 2006). Under normal conditions, tight junctions (TJs) maintain barrier integrity, but heat stress disrupts these structures, resulting in increased intestinal permeability or “leaky gut” (Horowitz et al., 2023). The pathology is marked by broadening of the villous stroma, localized necrosis, edema, and vascular congestion, along with increased apoptotic cell death in the lamina propria and infiltration by inflammatory cells (Rebez et al., 2023). A critical emerging concept in heat stroke pathogenesis is the involvement of the gut–brain axis and alterations in gut microbiota (Wen et al., 2021; Yi et al., 2021). The disrupted intestinal barrier permits the translocation of microbial toxins (LPS) into systemic circulation, triggering a profound immune response through activation of monocytes, macrophages, as well as endothelial cells, thus promoting a cytokine surge and contributing to systemic inflammation and multi-organ dysfunction (Ghulam et al., 2022; Lian et al., 2020). The microbial products can also cross the blood brain barrier and induce neuroinflammation and CNS dysfunction reflecting the involvement of the gut-brain axis (Wen et al., 2021; Yi et al., 2021). In fatal cases, intestinal necrosis and hemorrhage are often observed, with upregulated tissue factor expression in the intestinal mucosa, which initiates coagulation and formation of microthrombi (extrinsic pathway) (Sun et al., 2024).

Heat-induced gut dysbiosis is characterized by a reduction in beneficial bacterial populations such as *Lactobacillus* and Bifidobacterium, along with an overgrowth of opportunistic pathogens (Armstrong et al., 2018; Fu et al., 2023). Heat also alters the fungal microbiota, favoring proliferation of opportunistic fungi such as *Candida* albicans and Malassezia, particularly under conditions of immune suppression and epithelial injury (Sun et al., 2024; Pérez, 2021; Naik et al., 2025; Caetano et al., 2023). These fungi exacerbate mucosal inflammation and increase the risk of sepsis. Furthermore, oxidative stress and impaired host metabolism enhance susceptibility to fungal invasion, which triggers T helper 17 responses, which promote neutrophil recruitment, thus amplifying mucosal damage (Paoli and Cerullo, 2023; Valand and Girija, 2021). Moreover, the use of antimicrobial agents may further disrupt the bacterial-fungal balance, promote fungal virulence and suppress protective microbial functions (Krüger et al., 2019; MacAlpine et al., 2023; Lapiere and Richard, 2022).

**FIGURE 4:**
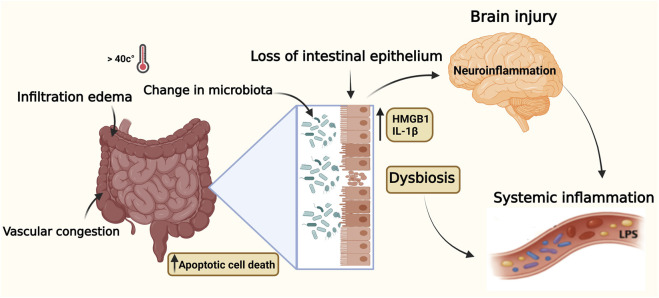
Illustration of elevated core body temperature effects on the gut-brain axis during heat stroke. Key inflammatory mediators (LPS, HMGB1, IL-1β) contribute to gut barrier breakdown and neuroinflammation. Pathway supported by human and animal evidence. Data illustrated in this figure are supported by human clinical data and complementary experimental animal studies. Created with BioRender.com.

Figure 4 shows that alterations in the gut-brain axis and intestinal microbiota contribute to systemic and neuroinflammatory responses observed in heat stroke pathology.

Beyond acute intestinal barrier failure, accumulating evidence indicates that heat-induced microbial translocation initiates a sustained inflammatory signaling axis linking the gut, endothelium, and brain. Circulating lipopolysaccharides and heat-induced damage-associated molecular patterns (particularly HMGB1) engage endothelial and immune receptors such as Toll-like receptor-4 (TLR4) and the receptor for advanced glycation end products (RAGE), activating NF-κB–dependent cytokine programs and promoting endothelial glycocalyx disruption and blood brain barrier permeability (Wang et al., 2025; Yan et al., 2006; Zhang et al., 2024; Yang et al., 2002; Leon and Helwig, 2010; Yoneda et al., 2024). This receptor-mediated cascade amplifies microglial activation and neuroinflammation, extending CNS dysfunction beyond the acute phase. Importantly, recent work suggests that gut-derived inflammatory mediators and dysbiosis—bacterial and fungal—may persist during convalescence, contributing to reported long-term cognitive and motor sequelae in heat-stroke survivors (Lawton et al., 2019; Yoneda et al., 2024; Walter and Carraretto, 2016). Besides, repeated or seasonal heat sensibility and aging seem to catalyze intestinal permeability, immune malfunction and pathogen predisposition (Roy et al., 2025). The effects of extreme heat and weather conditions, as well as environmental pollution, may compromise the natural and adaptive immune system, exposing the body to autoimmune and chronic inflammatory disorders (Rio et al., 2024; Imberti et al., 2025). These findings support the growing evidence that the gut epithelium-microbiota axis is a promising target for early detection and therapeutic intervention in heat-related illness.

#### 4.1.5 Neuroinflammation

The CNS is particularly susceptible to heat-induced injury due to its high metabolic demands and limited regenerative capacity. Neuronal damage results from the collective effects of many factors that include direct thermal stress, systemic inflammation, disruption of the blood brain barrier (BBB) and oxidative stress (Yoneda et al., 2024). Systemic inflammation is a key contributor to heatstroke pathogenesis and development of neuroinflammation. Heat stress triggers the release of damage-associated molecular patterns (DAMPs), such as HMGB1 and extracellular histones, which activate pattern recognition receptors (e.g., TLRs and inflammasomes). This induces the release of inflammatory cytokines, which disrupt the BBB, activate glial cells, and promote neuroinflammation and neuronal apoptosis (Yoneda et al., 2024). Neuroinflammation is a major contributor to central nervous system (CNS) morbidity and mortality during heatstroke (Walter and Carraretto, 2016). The elevated level of the inflammatory cytokine IL-1β in heatstroke cases, along with improved survival following its block, clearly indicates its key role in the pathogenesis of neuroinflammatory responses (Leon and Helwig, 2010; Zhang et al., 2021). Similarly, other cytokines such as IL-6, TNF-α, and IL-18 have been implicated in the pathogenesis of CNS damage (Kamidani et al., 2021; Leon, 2010; Hifumi et al., 2018). On the other hand, protective factors such as the anti-inflammatory cytokine IL-10, heat shock proteins, and pharmacologic agents including cytokine inhibitors and IL-1 receptor antagonists offer promising anti-inflammatory effects. In addition, mesenchymal stem cells (MSCs) have shown potential neuroprotective effects by reducing inflammation and preserving brain structures affected by heat (Zhang et al., 2020).

One of the earliest manifestations of systemic inflammation in heatstroke is the disruption of the blood brain barrier (BBB). The high temperature increases vascular permeability, allowing peripheral inflammatory mediators, immune cells, and microbial toxins such as lipopolysaccharide (LPS) to infiltrate the brain parenchyma (Kamidani et al., 2021; Leon, 2010; Hifumi et al., 2018). This BBB disruption permits the entry of neurotoxic substances and facilitates central nervous system injury through mechanisms involving the gut-brain axis.

In parallel, hyperthermia induces oxidative stress by impairing oxidative phosphorylation and increasing reactive oxygen species (ROS), leading to mitochondrial dysfunction, synaptic disruption, and neuronal cell death via apoptosis and necrosis.

### 4.2 Comparative appraisal of mechanistic pathways and their clinical relevance

Although the above mechanisms (systemic inflammation, coagulopathy, intestinal permeability, and neuroinflammation) collectively contribute to heat-stroke pathophysiology, the strength of supporting evidence and degree of clinical applicability vary. Amon these, systemic inflammation and coagulopathy have the strongest human clinical evidence, supported by consistent elevations in certain cytokines (IL-6, TNF-α and HMGB1) and routine coagulation abnormalities (D-dimer, PT/aPTT and thrombocytopenia), which showed valuable results in patient monitoring, risk stratification and prognosis in the acute care settings (Zhao et al., 2021; Iba et al., 2023a; Schlader et al., 2022; Leon and Bouchama, 2015). Several recent studies on heat stroke have also reported that neuroinflammation, acute neurological dysfunction and persistent cognitive sequelae, may be reflected by biomarkers such as S100β; however, this remains an emerging clinical tool rather than routine practice (Lawton et al., 2019; Yoneda et al., 2024; Walter and Carraretto, 2016). On the other hand, intestinal barrier disruption and gut-brain axis involvement, while supported by robust experimental and early human biomarker studies, still have limited clinical application (Wen et al., 2021; Ogden et al., 2020; Ghulam et al., 2022; Sun et al., 2024). Therefore, systemic inflammation and coagulopathy represent the most clinically applied mechanisms, whereas gut-barrier dysfunction and neuroinflammation are emerging domains. A comparative summary of evidence strength and clinical relevance for the above mechanisms is provided in Table 1.

**TABLE 1:** Comparative evidence and clinical relevance of key heat-stroke pathophysiological mechanisms.

Mechanistic domain	Main biomarkers	Clinical relevance	Rationale
Systemic inflammation	IL-6, TNF-α, HMGB1	High	- Central cause of severity and multi-organ damage. - Routinely monitored inflammatory markers.
Coagulopathy	D-dimer, PT/aPTT, platelets	High	- Biomarkers/tests are used for clinical monitoring and ICU care. - Have prognostic significance.
Neuroinflammation/CNS injury	S100β	Moderate	- Strong clinical phenotype. - Biomarker use is growing.
Intestinal permeability/gut-brain axis	Intestinal Fatty Acid–Binding Protein (I-FABP)	Emerging	- Evidence is supported by experimental and early human studies. - I-FABP is emerging as a marker of intestinal epithelial injury.

## 5 Neurological effects of heat stroke

It is believed that the central nervous system (CNS) is particularly vulnerable to elevated body temperature, with regions such as the cerebellum, basal ganglia, anterior horn cells, and peripheral nerves being especially affected (Guerrero et al., 2013). Heat stroke can profoundly affect the central nervous system, leading to both immediate and long-term neurological complications (Lawton et al., 2019; Yang et al., 2017).

### 5.1 Acute neurological complications

Acute brain dysfunction is a hallmark of heat stroke and may present as agitation, cognitive impairment, delirium, seizures, cerebellar ataxia or altered consciousness, resulting from a combination of hyperthermic neuronal injury, blood brain barrier breakdown, cerebral edema, and endotoxin-mediated neuroinflammation (Jarcho, 1967).

Cognition is defined as the set of mental abilities and processes that include memory, knowledge, attention, reasoning, problem solving, and comprehension. While its exact anatomical basis is unclear, it likely involves complex interactions across various brain regions including the cerebellum (Buckner, 2013). Hyperthermia, even if mild or brief, can impair memory (Racinais et al., 2008), attention, and information processing (Sun et al., 2011), sometimes permanently (Sun et al., 2012). Some of the cognitive processes may be affected by hyperthermia more than others. Short-term memory processing, for example, may be more affected than attentional processes. Cognitive effects may appear shortly after heat exposure ends and are linked to changes in brain connectivity (Stubblefield et al., 2013), particularly in regions like the limbic system and prefrontal cortex (Lee et al., 2012). Hyperthermia-induced short-term memory changes can be detected using electroencephalography through event-related potentials (ERPs), specifically mismatch negativity (MMN). Studies show that just 1 hour of heat exposure significantly reduces MMN (Sun et al., 2011), indicating impaired auditory memory formation and supporting observed declines in short-term memory. Although hydration may mitigate some effects, dehydration likely contributes to impairment (Sun et al., 2011). Cognitive decline affects both young and old, though baseline performance differs (Schlader et al., 2015). Most patients recover, but some may experience lasting cognitive or personality changes, ranging from mild deficits to severe dementia (Laxe et al., 2013; Sajatovic and Munshi, 2005).

### 5.2 Long-term neurological sequelae

Heatstroke leads to significant neurological damage, particularly affecting brain regions like the hypothalamus, cerebellum, cerebral cortex, Thalamus and hippocampus (Lawton et al., 2019; Leon and Bouchama, 2015; Yoneda et al., 2024). Even after apparent recovery, many patients develop long-term neurological sequelae that significantly impair quality of life, and many survivors develop persistent complications.

The cerebellum is especially sensitive, with early Purkinje cell loss observed in both imaging and autopsy studies, which often presents with long-term irreversible motor symptoms such as ataxia, dizziness, and nystagmus, and is visible as cerebellar atrophy on imaging (Yoneda et al., 2024; Malamud et al., 1946a). These changes are linked to increased inflammatory response and diffuse Purkinje cell coagulation and degeneration (Li et al., 2015). However, early signs of such damage are difficult to detect, thus delaying treatment and worsening the outcomes.

The hypothalamus, while being the body’s thermoregulatory center, is vulnerable to heat-induced damage. Edema and hemorrhage have been reported, and neuroinflammation mediated by elevated cytokines such as TNF-α and IL-1 contributes to its dysfunction, leading to impaired thermoregulation (Boucha et al., 2002; Yoneda et al., 2024; Malamud et al., 1946b).

The cerebral cortex begins to show abnormal changes after 24 h. Findings include neuronal loss, glial proliferation, edema, congestion, and microvascular and synaptic damage (Yoneda et al., 2024; Sharm et al., 1991). These injuries are driven by hemorrhages, oxidative stress, mitochondrial dysfunction, and inflammatory cytokines (Yoneda et al., 2024; Gao et al., 2024). After few days, other brain regions like the hippocampus and the thalamus may show MRI changes that include ischemia and inflammatory injury (Yoneda et al., 2024; Ba et al., 2005; Zhu et al., 2023). Damage to the hippocampus causes memory deficit, while thalamic lesions result in altered sensory processing and impaired consciousness. To optimize patient outcome, management of heat stroke survivors should include neurological rehabilitation, which encompasses cognitive training, physical therapy, and the use of neuroprotective medications.

## 6 Biomarkers for early detection and prognosis of heat stroke

While a core temperature above 40 °C is a key feature of heat stroke, it is not diagnostic in isolation, and lower temperatures do not exclude the condition. Given this limitation, increasing emphasis has been placed on biomarkers that can aid in early detection and prognosis of heatstroke (Schlader et al., 2022). Several biomarkers have been explored for this purpose. These include the following:

Muscle Damage (Schlader et al., 2022; Nybo et al., 2013)

Creatine kinase (CK)

Myoglobin

Cardiac Injury (Schlader et al., 2022; Bao et al., 2024)

High-sensitivity troponin I (hs-cTnI)

Coagulation Disturbance/DIC Risk (Iba et al., 2022; Bouchama et al., 1996; Abderrezak Bouchama et al.)

D-dimer

Thrombin–antithrombin III complex (TAT)

Plasmin–α2-antiplasmin complex (PAP)

Platelet count, PT, aPTT

Systemic Inflammation (Schlader et al., 2022; Mohyuddin, 2019)

Interleukin-6 (IL-6)

Tumor necrosis factor-alpha (TNF-α)

High-mobility group box protein 1 (HMGB1)

CNS Damage (Yoneda et al., 2024; Li et al., 2020)

S100 beta (S100β)

Neuron-specific enolase (NSE)

Renal Injury (AKI) (Kulasooriya et al., 2021; Goto et al., 2023)

Neutrophil gelatinase-associated lipocalin (NGAL)

Urinary NGAL (UNGAL)

Kidney injury molecule-1 (KIM-1)

Hepatic Injury (Schlader et al., 2022; Ji et al., 2021)

Alanine aminotransferase (ALT)

Aspartate aminotransferase (AST)

Intestinal Barrier Injury (Ogden et al., 2020; Ji et al., 2018; Wallett et al., 2021)

Intestinal fatty acid binding protein (I-FABP)

Endothelial Dysfunction/Glycocalyx Shedding (Schlader et al., 2022; Peng et al., 2023)

Von Willebrand factor antigen (vWfAg)

Soluble thrombomodulin

Syndecan-1

Several additional biomarkers have been investigated in experimental and critical care settings to enhance understanding of heatstroke pathophysiology. These include markers of intestinal injury (Cyptdin-2), renal stress (IGFBP-7, TIMP-2), vascular tone (nitric oxide, endothelin-1), leukocyte trafficking (soluble ICAM-1, E-selectin, L-selectin), and endothelial integrity (hyaluronan, circulating endothelial cells) (Iba et al., 2022; Schlader et al., 2022). While promising, their clinical utility remains limited and requires further validation.

To enhance their application in clinical settings, these biomarkers can be considered across three practical tiers based on current evidence. Routinely available but nonspecific markers (e.g., creatine kinase, troponin, D-dimer, PT/aPTT) support early recognition and severity assessment but lack heat-stroke specificity. Biomarkers with growing evidence (e.g., HMGB1, S100β, NGAL, I-FABP) demonstrate prognostic potential, but their use is limited by inter-study variability and the absence of standardized clinical cut-offs. Experimental and research-phase indicators, including glycocalyx injury markers (e.g., syndecan-1 and hyaluronan), and indices of endothelial repair (e.g., endothelial progenitor cell counts), currently provide insight into pathophysiology but require further validation before bedside adoption. This classification not only clarifies current clinical utility but also highlights promising pathways for future diagnostic and prognostic integration in heat-stroke management.

## 7 Artificial intelligence applications in heat stroke prediction and diagnosis

**FIGURE 5:**
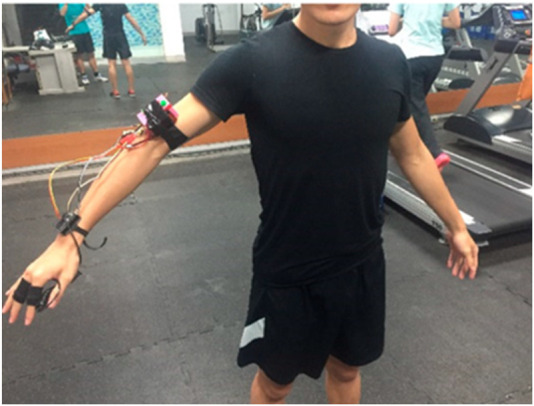
A photograph of the WHDD on the human arm. Reproduced from: Lin S-S, Lan C-W, Hsu H-Y, Chen S-T. Data analytics of a wearable device for heat stroke detection. Sensors. 2018; 18(12):4347. Licensed under CC BY 4.0 (Lin et al., 2018).

The use of artificial intelligence (AI) and machine learning (ML) in the early detection and management of heat stroke is an emerging area of research. Such systems integrate multiple sensors to track key parameters, including heart rate, core body temperature, ambient temperature, and relative humidity (Son et al., 2021; Lim et al., 2024; Hosen et al., 2025). They process these inputs through a fuzzy logic controller to calculate heat stroke risk level to trigger alerts under hazardous conditions. Data are transmitted via the Internet of Things (IoT) to a ThingSpeak server and visualized on an Android application, providing both numerical and graphical outputs during both rest and activity (Son et al., 2021; Lim et al., 2024; Hosen et al., 2025). These tools can enable early recognition of heat stroke before appearance of clinical signs and can distinguish heat stroke from other life-threatening conditions (Son et al., 2021; Lim et al., 2024; Hosen et al., 2025). They can also guide immediate clinical decisions. Furthermore, predictive models have been designed to evaluate disease severity and enable personalized management based on predicted complications. One notable example is a wearable IoT-based heat stroke detection device designed for real-time risk monitoring (Teo, 2020). Another example is a wearable heat detection device (WHDD) that monitors heart rate and core body temperature to predict heat stroke risk level (Figure 5) (Lin et al., 2018). Another innovation involves a wearable vital-sign monitoring device that applies supervised machine learning algorithms such as k-nearest neighbors (KNN0 to create a personalized heat stress index (Shimazaki et al., 2022). These innovations are quite useful as preventive tools for high-risk individuals who are exposed to high-temperature environments such as athletes, military personnel, field workers and patients in emergency settings.

A special innovation designed for firefighters and outdoor workers is a wearable device fabricated with a flexible sensor network using inkjet-printed nano-functional inks on a semi-permeable substrate (Maroli et al., 2024). This battery-free system enables real-time monitoring of skin temperature, humidity, and muscle contraction through wireless near-field communication (NFC) for data transmission to smartphones. The sensors are integrated into a bioinspired adhesive membrane, ensuring strong skin conformity and stable data collection (Maroli et al., 2024).

Most systems remain at prototype or lab-validation stages, underscoring the need for rigorous clinical trials and real-world evaluations to determine their reliability and clinical utility in early heat-stroke detection and risk stratification. A pilot study was conducted to examine research grade wearable sensors of heat-strain among outdoor agricultural workers in a natural hot environment (Kwaro et al., 2025). The system was able to estimate core temperature, heart rate, and environmental heat index and activity levels continuously during a 14-day period of monitoring with high acceptability (>95%) and high data completeness to use in estimating core-temperature. Nevertheless, the research also revealed inconsistent precision of non-core temperature sensors, poor transparency of the algorithms, unverified reproducibility in different settings, and the lack of regulatory channels to certify the medical implementation. These results demonstrate that despite the promising potential of wearable platforms to be used as an early indicator of heat-strain, additional validation, standardization and regulatory assessment are necessary before they can be applied to a more regular clinical or occupational safety practice. More work needs to be done to overcome the accuracy limitations, data privacy, algorithm transparency, and safety issues.

Table 2 summarizes different wearable and AI-based heat-stroke detection technologies and their current validation status.

**TABLE 2:** Validation status of wearable heat-stroke detection technologies.

Device	Current status	Supporting reference (No.)
Wearable Heat Detection Device (WHDD)	Human-tested (pilot validation)	Lin et al. (2018)
IoT body-temp + HR + humidity sensors	Human-tested (pilot validation)	Normahisham and Yahya Atan (2023)
Machine-Learning–Based Wearable (KNN model)	Human-tested (pilot validation)	Shimazaki et al. (2022)
Inkjet nano-functional biosensing patch	Prototype (engineering validation)	Maroli et al. (2024)

## 8 Management of heat stroke

Heat stroke is a life-threatening medical emergency requiring immediate diagnosis and prompt treatment. Effective management begins with rapid recognition, ideally guided by environmental awareness, elevated core body temperature (≥40 °C) measured from the rectum, and central nervous system dysfunction (Rublee et al., 2021). Early empirical treatment is advised as it improves survival rate and prevents long-term complications (Rublee et al., 2021). Initial steps include removing the patient from the hot environment and initiating external cooling while ensuring patent airway and stabilized breathing, and circulation (Chan and Mamat, 2015). The cornerstone of treatment is prompt body cooling, with cold water immersion being the most effective and evidence-based method, with a cooling rate up to 0.35 °C/min (Rublee et al., 2021;Chan and Mamat, 2015). Alternative strategies include evaporative cooling (mist and fan), ice packs to major vessels, and cooling blankets. In severe or refractory cases, internal cooling methods such as gastric or bladder lavage, intravenous cold fluids, and endovascular cooling devices may be used (Rublee et al., 2021; Chan and Mamat, 2015). Benzodiazepines can be used to control shivering, and deep sedation with neuromuscular blockade may be considered to reduce metabolic heat production in selected patients (Rublee et al., 2021). In addition, patients should receive general supportive care of critically ill patients that involves continuous monitoring of vital signs, fluid balance, urine output, electrolytes, coagulation status, renal status and hepatic function (Rublee et al., 2021). Acute complications such as rhabdomyolysis-induced acute kidney injury and hepatic failure should be identified and promptly managed to prevent further deterioration (Chan and Mamat, 2015). Innovations, along with strengthened health systems and educational programs to increase awareness of high-risk populations are essential to improving outcomes (Rublee et al., 2021; Chan and Mamat, 2015).

## 9 Conclusion and future directions of research

Heat stroke is a life-threatening emergency defined by rise in core body temperature ≥40.0 °C, accompanied by acute central nervous system (CNS) dysfunction and often complicated by multi-organ failure. It is caused by the combined effects of thermoregulatory failure, systemic inflammation, coagulopathy, and direct heat-induced cellular damage. The current article describes the pathophysiology of heat stroke, highlighting the roles of endothelial dysfunction, gut-brain axis disruption, neuroinflammation, and immune activation. It also discusses potential methods of early detection, including the biomarkers of organ injury and the predictive wearable devices that incorporate AI or ML technologies. Standard management includes rapid cooling and supportive care; however, personalized treatment and proper management of complications are necessary. Long-term follow-up is necessary to determine residual disabilities of the survivors, and to guide the development of comprehensive rehabilitation programs aimed at improving their quality of life.

In spite of the significant improvements in management and early detection of heat stroke, there are still many clinical and research gaps to be addressed. Future research needs to focus on biomarker-based diagnostic strategies and the development of wearable predictive technologies. These tools must be standardized and integrated into routine practice. Combining biomarker profiles with artificial intelligence (AI) tools may enhance pre-hospital triage and individualized treatment strategies, particularly in high-risk settings. In addition, more novel cooling methods (both pharmacological and non-pharmacological) should be considered to accomplish faster and more effective cooling. The combined contribution of translational research, AI technology and public-health interventions that include occupational heat-safety policies and expansion of urban green spaces will all advance early detection, prevention, and long-term recovery in individuals affected by heat stroke.
